# The Struggle to Regulate Precarious Work Arrangements to Minimize Their Adverse Effects on Health and Safety in Australia

**DOI:** 10.1177/27551938241234753

**Published:** 2024-02-25

**Authors:** Elsa Underhill, Michael Quinlan

**Affiliations:** 1 2104 Department of Management, Deakin University, Burwood, Victoria, Australia; 2 School of Management and Governance, 7800 University of New South Wales, Sydney, NSW, Australia

**Keywords:** occupational health and safety, employment regulation, precarious work, trade unions

## Abstract

As in other countries, the growth of precarious work arrangements in Australia from the late 1970s has had significant adverse effects on occupational health and safety (OHS). While there is now a large body of global research on this issue and its connection to the rise of neoliberalism, there has been less investigation of efforts to address these problems. This article reviews regulatory interventions in Australia over the past two decades. It particularly focuses on industrial relations regulation, which can play a critical role in addressing at least some of the underlying reasons why precarious work undermines OHS. The most significant of these changes were passed by the Australian Parliament in February 2024, including a highly controversial but world-leading creation of minimum standards for platform workers.

While precarious work arrangements date back for centuries, they expanded rapidly from the mid-1970s, coinciding with the rise of neoliberalism. From the 1980s (in trucking) and the mid-1990s more generally, a growing body of research and government inquiries in Australia (on maritime work, agency work, home-based work, road transport, and insecure jobs) uncovered the adverse health and safety effects of various categories of precarious work. By the 2000s evidence was compelling and matched research being undertaken in many other countries.^1^ Several models have been proposed to explain how precarious work undermines occupational health and safety (OHS). The PDR model (see, for example, Underhill and Quinlan^2^) argues that three characteristics typical of precarious work undermine OHS, namely more intense economic/financial and reward pressures (including incentive payment plans and irregular income), disorganization (poorer induction, training, and complexities typified by multi-tiered subcontracting), and regulatory failure (weakened protection/enforcement or bypassing of minimum standards like wages and hours). One corollary of this model is that addressing these problems requires measures that reshape labor standards in addition to OHS laws in ways that restrict the capacity to exploit precarious workers and thereby also reduce the incentive to use them.

For much of the twentieth century, Australia had a centralized industrial relations regulatory framework whereby wages, hours, shift breaks, penalty rates for temporary workers, and other working conditions were laid down in legally enforceable “awards” covering a particular occupation or industry. Almost all employees (including those in nonunion workplaces) were covered by these awards, which were made and updated by union-employer negotiation, or if agreement failed, through compulsory arbitration by the Australian Industrial Relations Commission (now Fair Work Commission [FWC]).

This encompassing framework operated alongside OHS and workers’ compensation laws. As in many other countries, under the sway of neoliberalism from the 1980s both union density and this regulatory regime weakened, undermining OHS especially regarding precarious workers.^3^ A growing group of workers not deemed employees but rather self-employed (including digital platform workers) had no protection under industrial relations or workers’ compensation laws. Nonetheless, the election of a federal Labor Party government in 2022 led to several measures gradually enacted that aimed at better protecting precarious workers. The government does not, however, have a majority of members in the Senate (house of review), and every legislative change is subject to negotiations with minor political parties and independent members of parliament. This has slowed but not halted the process of regulatory change. In December 2023, the Australian Parliament passed legislation to enable temporary agency workers to be paid the same rate of pay as host workers; and in February 2024, the Australian Government passed new world-leading minimum standards for gig economy workers and owner-drivers in the transport industry, and legislated new pathways to permanent employment for casual workers.

This article will briefly describe efforts to better protect precarious workers from 2000 to 2023. The first section outlines the context for recent regulatory proposals to curtail the further erosion of OHS and employment standards for precarious workers in Australia. The second section provides an overview of the development of the *safe rates* concept in road transport and setting minimum rates for gig/platform workers. In the third section, emerging regulation of labor hire employment and the introduction of minimum wage rates for harvest workers, a group dominated by foreign workers on temporary visas, is examined. These areas of precarious work have proven problematic to regulate in other countries. The conclusion highlights how the Australian experience mirrors overseas challenges while also providing evidence of more effective interventions, virtually all driven by union campaigns.

## Context for A Shift in Regulatory Arrangements Protecting Precarious Workers

By the early 2020s, the Australian labor market showed clear signs of stagnation accompanied by growing income inequality. Two decades of conservative neoliberalist policies had weakened unions and diminished union membership to the extent that collective action rarely occurred.^4^ Wage growth lagged significantly behind productivity growth, and real incomes for the top quintile of households was growing at almost twice the rate of those in the lowest quintile.^5^ The OECD aptly described these changes as “inclusiveness has been eroded”.^5^ Most disturbingly, a falling unemployment rate was not producing wage growth.^6^ High among the explanations for these outcomes was the withdrawal of government support for institutions that supported workers’ rights, notably trade unions and the FWC.^5,6^ Pennington,^7^ for example, estimated the proportion of private sector workers covered by collective agreements had fallen to around 12 percent by 2018. Also significant was the continuing expansion of precarious employment, including underemployment; Stanford^4^ estimated one quarter of the workforce was employed on a casual basis in 2018, and nonstandard employment in total accounted for over half the workforce. A Senate inquiry into job insecurity that commenced in 2020 produced a substantial report in 2022 that highlighted many of these problems, including interim reports focused solely on low wage and employment concerns in aged care, platform work, and labor hire employment.^8^

The outbreak of the COVID-19 pandemic in early 2020 magnified the problems of precarious work and publicly revealed the low wages and precarious employment conditions of many workers now regarded as essential workers. COVID-19 demonstrated the health vulnerabilities of societies heavily dependent on precarious work arrangements or a large informal sector. Close interconnections between poor public health and precarious work arrangements, including the spread of infectious disease, well known via government inquiries and research since the late nineteenth century, were “rediscovered”.^9^ Precarious employment was especially prevalent in sectors like food production, warehousing/logistics, retailing, aged care facilities, and transport/delivery, which had to operate even during lockdowns. These workers were often low-paid and lived in crowded households, exacerbating the risk of disease spreading, especially given an absence of sick leave—which discouraged reporting symptoms. In Australia the subcontracting out of security in quarantine hotels was responsible for at least one major outbreak. Disease also spread through multiple jobholding (including through aged care facilities) by workers to increase an otherwise low income or to compensate for irregular shifts. State and federal governments sought to fill this gap by prohibiting multiple jobholding for select occupations and establishing COVID-19 isolation payments for those unentitled to sick leave.

In a rare admission, the premier of the state of Victoria (Daniel Andrews) labeled precarious work as toxic and pledged to do something about it. Among other things, Victoria has maintained special payments to 400 specified occupational groups of workers who, as temporary workers, were not entitled to sick leave. However, as elsewhere with isolated exceptions—Germany legislated to severely restrict agency workers and subcontracting in meat works—the lessons of the pandemic were rapidly lost after mass vaccination was achieved by many countries including Australia, and media lost interest even though COVID-19 continued to mutate and kill people, if not at the original scale. An important exception was a Senate inquiry into job insecurity, whose request for submissions made specific reference to COVID-19 connections/implications and took this up in its final report.^10^ This report and that on road transport helped inform a raft of industrial relations reforms introduced in 2022 and more especially, in 2023 by a newly elected Labor government.

The 2022 federal election campaign by the Australian Labor Party focused substantially upon government policies to counter the stagnation in wages and growth in precarious employment that had become the focus of public debate over 18 months of the pandemic. These reforms included supporting wage increases for low paid workers, rebalancing the composition of members of the FWC to better reflect worker interests, promoting collective bargaining, and introducing new rights for platform work and casual employees.^11,12^ Following considerable engagement with experts and interest groups, in September 2023 the federal government introduced a “closing the loopholes” industrial relations bill that included an array of significant provisions affecting those in precarious work. The bill significantly increased the penalties (including jail time) for wage theft by employers. This bill was designed to deal with instances of widespread nonpayment of minimum wage entitlements, especially to temporary workers, including those working for large corporations in service stations, retail, and universities. Another set of provisions provides for the setting of minimum rates for platform/gig workers by the FWC. There were also provisions dealing with temporary workers, enabling those working for an employer for over a year to transition to permanent/ongoing positions (and acquire access to sick, annual, and long service leave). New provisions ensuring temporary agency/labor hire workers received the same payment as direct employees have been especially contested. Among others, the mining industry strongly opposed the same job, same pay principle. At the same time, contracting and labor hire posed a serious OHS challenge in the industry. For example, the Board of Inquiry into a methane explosion at the Grosvenor coal mine in Queensland in May, 2020, which severely burned five workers (all labor hire), found these workers were reluctant to raise safety concerns for fear of losing their job (labor hire workers can be removed without cause). Further, the third-party arrangement created greater ambiguity in terms of OHS management at the mine—similar problems have been identified at other mines and in other industries. Prior to the incident the entire mining workforce had been labor hire; afterward, the company converted to a directly hired workforce.

These proposals, after extensive industry consultation and negotiations with minor political parties and independents, were finally passed by the Australian Parliament in December 2023 and February 2024. They represent a major break from recent decades of neoliberalism and offer the potential to slow the growing levels of inequality and declining real wages that have characterized the Australian workforce since the 1980s. In the following sections, we outline the extended campaigns which have contributed to this path-breaking shift in labor regulation for precarious workers.

## The Struggle to Regulate Precarious Work 2000–2023: Road Transport and Platform Delivery Services

In the mid- to late-1990s the Textile Clothing and Footwear Union (TCFU) and Transport Workers Union (TWU) initiated campaigns to protect home-based garment makers and long-haul self-employed truck drivers, both predominantly nonunionized and recently arrived immigrants in the case of garment workers. These were longstanding areas of precarious work (stretching back well over a century in the case of home-based garment makers) but had grown since the 1980s due to the competitive pressures of clients and use of elaborate supply chains (multi-tier subcontracting) to drive down labor costs.

For both groups of workers, the combination of low wages (piecework or kilometer-based payment) and long hours—what was once termed *sweated labor*—was not merely exploitative but had adverse effects on their OHS (see, for example, Mayhew and Quinlan^13^). The Fairwear campaign regarding homeworkers garnered significant public support, aided by union-sponsored media coverage and a series of government inquiries that exposed the problems. In 2013 federal legislation was enacted, setting minimum wages and conditions for home-based workers with presumption of responsibility at the top of the supply chain and contractual tracking mechanisms and reporting obligations empowering the union to more effectively enforce these conditions. The legislation also mandated workers’ compensation coverage for these workers in the case of illness and injury.

Industrial and political struggle occurred in the parallel community of the road transport industry. The state government of New South Wales initiated an inquiry into the impact of commercial arrangements, including shipper pressures and multi-tiered subcontracting and supply chains on the OHS of truck drivers. This inquiry (which extended its investigation nationwide) identified a close connection between commercial pressures and kilometer-based payment of owner-drivers and poor OHS, arguing drivers needed to be paid a minimum *safe rate* to protect both them and the wider community. A review of evidence by the National Transport Commission^14^ confirmed the pay-safety connection, and in 2012 federal legislation was enacted to establish the Road Safety Remuneration Tribunal (RSRT), which could set minimum rates and conditions for owner-drivers who, as self-employed workers, were normally excluded from coverage by arbitration tribunals (i.e., the FWC). The RSRT made one ruling on waiting-time payments, but it was then subjected to a coordinated attack from neoliberalists and the conservative government elected in 2013, which abolished it in 2016.

Notwithstanding its demise, the RSRT left an important legacy. Similar safe rates legislation was adopted for some categories of truck drivers in South Korea, though this too lapsed after a change of government, notwithstanding a fierce industrial struggle. The TWU succeeded in having the safe rates concept adopted by the International Labour Organisation (ILO). The connection between low pay and poor safety and health outcomes was not a new idea, nor was it confined to trucking. Knowledge of the connection dated back over a century as highlighted by government inquiries into sweated labor from the 1890s and periodically reinforced by a series of studies of the OHS effects of piecework and subcontracting across a range of industries.^15–17^ The safe rates concept also aligned with the ILO's decent work agenda and its growing recognition of the importance of supply chains in terms of their OHS effects—something still belabored given strident resistance by employer representatives on the ILO. Finally, like its South Korean counterpart, the TWU has not abandoned its campaign for safe rates legislation in Australia and is pressing for another RSRT-like tribunal, something recommended by a Senate inquiry into road transport.^18^ In response, the Australian government has proposed the establishment of a road transport specialist panel within the FWC that will set minimum rates for truck drivers and hear disputes about unfair contracts in the road transport sector.^19^ This legislation, passed in February 2024, has received wide support from unions and employers within the transport sector, potentially resulting in greater longevity than the RSRT.

Approximately five years ago, the TWU also initiated a safety-based campaign on behalf of food delivery workers working for Uber and similar firms, which has widened to encompass other platform/gig workers. Food delivery operatives were predominantly composed of new immigrants or foreign workers on temporary visas; they were essentially engaged in an app-based subcontracting arrangement as purportedly independent contractors at low rates of pay, and were subject to considerable irregularity in work/hours. Highlighting several fatalities among food delivery workers, the TWU campaign focused on exploitation, poor safety, and the absence of workers’ compensation entitlements. As with truck-drivers, the union sought to establish safe rates in the form of enforceable minimum wages and conditions, and it pushed for them to be covered by the FWC. The federal government has proposed the introduction of a new category of workers, known as *employee-like* workers to empower the FWC to establish minimum rates of pay (not employment conditions) for platform/gig workers.^20^ These proposals have received majority support in Parliament and the legislation was passed in February 2024. 

## Regulating Labor Hire and the Establishment of A Minimum Wage for Harvest Workers

Labor hire or temporary agency employment has come under increased scrutiny in Australia over the past decade. It was initially associated with lower rates of pay and higher levels of workplace injury in traditional blue-collar industries such as warehousing, construction, and maintenance.^2^ In 2011, the Australian government responded with major regulatory reform to concerns about the growing incidence of workplace injuries and OHS regulatory complexities associated with labor hire employment. OHS responsibilities were amended to eliminate the distinction between host and employer through the introduction of the catch-all grouping “Persons Conducting a Business or Undertaking” (PCBU), and coverage was broadened by replacing “employee” with “worker,” thereby ensuring that hosts and labor hire operators held responsibility for the OHS of labor hire workers.^21^

Around the same time, labor hire operators began supplying harvesting labor in the horticulture sector, followed soon after by unskilled workers in the meat processing sector. Seasonal harvesting work became dependent upon young, temporary migrant workers (TMWs) following changes to visa arrangements in the mid-2000s. These young, often non-English speaking TMWs lacked familiarity with the Australian labor market and employment rights and were well suited to labor hire arrangements that could connect them with geographically dispersed farmers to enable completion of the minimum workdays required to extend their visa.^22,23^ TMWs employed by farmers were already low paid; labor hire companies’ expansion into horticulture simplified hiring by farmers while simultaneously cutting wages and providing substandard accommodation for workers. The United Workers Union (UWU; formerly National Union of Workers) sought to recruit these TMWs but struggled to gain traction with highly mobile, foreign workers with a short-term commitment to working in horticulture. Importantly, the UWU persuaded the Victorian branch of the Australian Labour Party to include labor hire licensing in their party platform.

In 2015, an ABC television documentary revealed the highly exploitative practices that had by then become commonplace across horticulture and meat processing, including the alleged sexual assault of young, female Asian workers. Around the same time, the Fair Work Ombudsman (the national labor inspectorate) prosecuted a major network of tiered subcontractors in poultry processing for TMW labor abuse.^24^ They also conducted two national investigations of employment practices in horticulture that identified substantial breaches of minimum employment standards.^25,26^ An Australian Senate inquiry followed. Their report was tellingly titled *A National Disgrace: The Exploitation of Temporary Work Visa Holders*. Temporary migrants were exploited to a much greater degree than previously known, and labor hire employers were at the forefront of that abuse.^27^

The then neoliberal government's response to the Senate inquiry was negligible. They established a Migrant Workers Task Force but implemented only one of the task force's recommendations—in relation to the responsibilities of franchisors.^28^ Other recommendations, such as the introduction of a national labor hire licensing scheme and the enhancement of a range of visa and employment rights specific to TMWs, were ignored. Three state-level Labor Party governments, in contrast, offered more targeted responses. Each established an inquiry into labor hire employment, resulting in the introduction of three state-based licensing regimes.^29–31^

The licensing requirements vary between states, but typically include evidence of directors being of good character and that the organization has not recently been prosecuted for OHS and employment law breaches. In Victoria, registration also requires the provision of information about the number of TMWs employed, their wages, and conditions of employment. A recent applicant in Victoria, for example, was refused a license because the wage rates paid breached the relevant minimum hourly rate of pay.^32^ While the state labor hire registration systems apply to a range of high-risk industries, prosecutions for breaches of licensing requirements have been dominated by suppliers of labor in horticulture, including hosts using unlicensed operators. In both Queensland and Victoria, around 10 percent of labor hire operators have been refused licenses or had those licenses canceled/suspended.

The impact of the licensing schemes upon employment standards has not yet been evaluated; however, the licensing agencies have taken a proactive approach, and the national government is developing a national licensing scheme to extend higher standards to all states. These schemes seek to place a floor under wages and employment conditions to reduce the pressures upon TMWs to work in unsafe and hazardous conditions. Furthermore, in December 2023 the national government passed legislation to provide for the same job, same pay principle to apply to labor hire workers relative to host workers. This is intended to prevent host employers from avoiding collective agreements with unions by outsourcing to labor hire operators. Its reach will extend well beyond horticulture to mining, airlines, and other more traditionally unionized sectors.

The most significant change to directly benefit TMWs in horticulture, however, occurred in 2021 with the introduction of a minimum wage for piece workers. Previously, harvest workers could be employed under hourly rates of pay or piece rates. In an extraordinary gap in labor regulation, the horticulture award provided that the “average competent worker” employed under piece rates could expect to earn 15 percent more per hour than the minimum hourly rate, but a minimum wage for piece workers was not guaranteed. Unsurprisingly, most piece workers earned well under the equivalent minimum hourly rate of pay, and even hourly paid workers were commonly paid less than the legal hourly minimum rate.^15,33^ Minimum rates were poorly enforced by an under-resourced labor inspectorate (especially given the geographic spread of horticulture across Australia), and the vagueness of the term *average competent worker* meant that employers unilaterally set piece rates. In addition, the short-term nature of seasonal employment for TMWs and language difficulties meant that TMWs were difficult to unionize and lacked an understanding of their employment rights.^34^

Harvesting work is arduous and can be high risk. It is typically undertaken in high summer when workers are exposed to extreme heat with the risk of sunstroke and dehydration, and it involves hard physical labor such as lifting/carrying of heavy bags of fruit and vegetables, continual bending to pick low growing crops, and climbing ladders to pick fruit from higher tree branches. TMWs paid piece rates, as well as those employed by labor hire, were most at risk. Underhill and Rimmer^35^ found they were more likely to delay taking fluids, working through higher temperatures, and taking greater risks with ladders and heavy lifting than those employed on hourly rates or hired directly by farmers.

In 2020, the Australian Workers Union (AWU) lodged a claim with the FWC to insert a minimum wage rate for piece workers in the horticulture award. The UWU later joined in the case. The case was contested by several national employer bodies and was heard before a full bench of the FWC. The AWU case drew primarily upon a national survey of harvest workers’ earnings conducted by Underhill and Rimmer,^35^ supported by individual worker testimony of extremely low piece work earnings. The UWU added to the case, drawing upon academic research by Howe and colleagues^26^ to provide qualitative evidence of the widespread use of untenably low piece rates. Employer associations counted with their own research, alongside farmers’ accounts of employment practices, but their research lacked the academic rigor of Underhill and Rimmer^35^ and Howe and colleagues,^26^ and it could not withstand scrutiny by the FWC. The FWC described the piece work clause as “not fit for purpose” and determined that all piece workers should receive a minimum rate of pay equivalent to the minimum hourly rate.^36^ This was a major win for harvesting workers and unions. The impact of this decision has not yet been fully evaluated; however, preliminary research suggests hourly rates are becoming more widespread in horticulture with some employers offering output-based bonuses that supplement rather than displace the minimum wage.

The introduction of a minimum rate of pay for harvest workers offers a significant financial and OHS benefit to workers no longer subject to the risks associated with working harder, faster, and cutting corners in a hazardous industry. This will benefit many TMWs. It does not, however, address other aspects of exploitation that TMWs experience across Australia; two groups are especially vulnerable.

The first group consists of workers employed under the Pacific Australia Labour Mobility Scheme. The number of Pacific Island workers employed under this program expanded when Australian borders closed to all other TMWs under COVID-19 lockdowns. Their employment is more heavily regulated than other TMWs in horticulture and food processing, but they can only be employed by the labor hire employers to whom they are tied. Like other TMWs, however, they are vulnerable to exploitation, notably through low wages and compulsory deductions for employment-related “training” and substandard accommodation. A major agriculture labor hire specialist, for example, was suspended from employing Pacific Islanders for 12 months following prosecution for underpayments. The second large group of TMWs consists of international students, employed on a temporary basis in jobs such as hospitality, cleaning, and online platform work in urban locations. Both groups are vulnerable to exploitation due to work restrictions associated with their visa status.

In 2023, the Australian government legislated to ensure TMWs have the full employment protections of the Fair Work Act.^37,38^ Further legislation targeting the intersection of employment and migration rights, such as protecting TWMs from deportation for breaching visa work requirements and penalizing employers who induce such a breach or coerce a TMW to accept exploitative conditions, have stalled in parliament.^39^

## Conclusion

In almost all countries separate bodies of protective laws regulate industrial relations/minimum labor standards, workplace health and safety, and workers’ compensation. Although there is some overlap, this divide is a historical overhang of legislative developments, and its limitations are especially manifest when it comes to precarious work arrangements. The low pay and irregular work that typify precarious jobs are health damaging, and, therefore, the remedy to these aspects lies more in the realms of industrial relations laws, not OHS law. Another limitation of the trifurcation of labor protection law is that in two of these areas, the legal protections apply to employees, not to the broader category of workers. This means employers can both exploit the lower entitlements and protections of temporary workers (including labor hire) or seek to evade industrial relations and workers’ compensation protections altogether via subcontracting/contracting, including app-enabled platform work. Indeed this bypassing of minimum standards is central to Uber and similar business models. The one body of law where such evasion is not possible is OHS legislation, especially in Australia since 2013 where, rather than referring to employers and employees, the law refers to the far wider concepts of persons in charge of a business or undertaking (which includes contractors, designers, suppliers, and indeed anyone influencing OHS) and workers. Yet even here, enforcing legislative duties in complex and dispersed work arrangements represents a major challenge to inspectorates tasked with this. Further, to date OHS regulatory requirements regarding precarious workers like food delivery workers have been fairly anodyne—with codes only referring to protective equipment and the like.^40^

This is why the industrial relations campaigns and reforms described in this article are important if the underlying causes of inferior OHS among precarious workers are to be effectively addressed. In several countries like the United States (or particular states like California), attempts have been made to bridge gaps in regulatory protections by redefining gig workers and others as employees. This has proven to be a fraught, long and difficult battle. In Australia an industrial relations framework marked by a centralized tribunal augmenting direct negotiations between unions and employers, though much diminished in scope and power since the early 1980s, still has the power to make wide-ranging determinations affecting even the vulnerable and non-unionized. This power is most evident with respect to establishing a minimum wage for piece workers in horticulture, where insurmountable obstacles prevented unions from recruiting members and acting collectively. Enhancing the FWC's role to ensure labor hire and direct hire workers doing the same job are paid the same (thereby removing the economic incentive to use labor hire) and covering groups of workers like gig workers—who were previously unprotected as purportedly self-employed—represents a potentially more effective strategy. Also important is recognizing the intersection of employment and migratory rights, where the risks of deportation undermine workers’ rights to access minimum employment standards. The Australian Government, following months of consultation and negotiation with key stakeholders, minor political parties and independent members of Parliament, has been able to achieve legislative change which markedly improves minimum standards for precarious workers, thereby creating a floor under the work pressures and low wages experienced by precarious workers. Some of these changes represent a ‘catch-up’ with international standards, whilst others, such as creating minimum standards for gig/platform workers, are a world first. In the longer term, these changes may provide a basis for extending workers’ compensation coverage as was done with home-based garment workers a decade ago. At least as important, this analysis provides a model and leverage point for legislative reform movements in other countries.
